# Thermoring basis for thermo-gated TRPV2

**DOI:** 10.21203/rs.3.rs-6049325/v1

**Published:** 2025-02-21

**Authors:** Guangyu Wang

**Keywords:** Cooperative unfolding, gating, noncovalent interaction, protein-lipid interaction, thermodynamic signature, thermoring structure, protein stability

## Abstract

The heat-responses of the homotetrameric thermosensitive transient receptor potential vanilloid (TRPV)1–4 channels are use-dependent. The initial short heat stimulus typically alters the temperature threshold and sensitivity for the subsequent one. The precise underlying structural motifs have not been identified except for TRPV1 and TRPV3. Since the release of lipid from the active vanilloid site is necessary for the initial heat activation of TRPV1 or TRPV3, the 3D cryo-EM structures of apo rat TRPV2 with or without any lipid in different gating states were analyzed using a highly sensitive thermoring model. The results indicated that two lipids in the voltage sensor-like domain and at the vanilloid site needed to be released to achieve theoretically and experimentally matched start and end thresholds and thermosensitivities during the first and second heat sensations. Therefore, this study further elucidated the role of lipids at various sites in the use-dependent heat responses of thermosensitive TRPV1–4 channels.

## Introduction

Heat-responsive homotetrameric thermosensitive transient receptor potential vanilloid 1–4 (TRPV1–4) channels are multi-domain integral membrane proteins. Each monomer consists of the first four transmembrane helices (S1–S4) forming the voltage sensor-like domain (VSLD) and the latter two (S5–S6) shaping the pore domain including two pore turrets and the pore helix between them. The S4–S5 linker is located at the active gating center, the transient receptor potential (TRP) domain links S6 with the C-terminal domain, and the pre-S1 domain connects the VSLD to the ankyrin repeat domain (ARD) (1).

These membrane proteins have specific start thresholds (T_th_) for heat activation. For example, 42°C for rat TRPV1 (rTRPV1), 48–52°C for rat TRPV2 (rTRPV2), 32–39°C for mouse TRPV3 (mTRPV3) and 25–35°C for TRPV4. However, their end thresholds (T_th_) typically range from 56°C to 61°C. In addition, except for heat-insensitive human TRPV2 (hTRPV2) (2–3), they exhibit a high temperature sensitivity Q_10_, which is the activity ratio of an ion channel assessed at temperatures 10°C apart. For instance, Q_10_ > 20 for TRPV1 and TRPV3, 10–19 for TRPV4, and Q_10_ > 100 for rTRPV2 (4–13).

It is worth noting that both mTRPV3) and rTRPV2) channels show use-dependent heat sensitization, while rTRPV1) and TRPV4 are characterized by use-dependent heat desensitization (4, 6, 8, 10, 14–20). When the complex protein-lipid interactions regulate their temperature responses, the role of the specific lipid in temperature-evoked gating transitions needs to be precisely defined (21–22).

Although temperature-dependent cryogenic electron microscopy (cryo-EM) structures of rTRPV1 and mTRPV3 have been captured (23–24), and their thermodynamic signatures for specific T_th_ and Q_10_ have also been revealed by the recently-developed high-sensitive thermoring model (25–31), much less is known about the origins of the high T_th_ and Q_10_ of rTRPV2. Since the release of a vanilloid lipid phosphatidylinositol (PI) or phosphatidylcholine (PC) is required for heat activation of TRPV1 or TRPV3 (23–24, 28–29, 32), several cryo-EM structures of rTRPV2 with or without phosphatidylethanolamine (PE) lipids at the active vanilloid site and in the VSLD were investigated in this study using such a thermoring model (33–35).

Once the initial pre-open closed state with PE bound only in the VSLD was identified with a calculated melting temperature threshold (T_m_) of 50°C to match the experimental start T_th_ of 48–52°C (17, 36–38), it was used as a control to define other gating states with the theoretical and experimental thermodynamic parameters matched. For example, the PE-free open state with a matched end threshold of 56°C and matched structural and functional thermosensitivities (Ω_10_ and Q_10_, respectively) of 154.6 for the first heat activation (17, 34–38), the second PE-free pre-open closed state with a matched start threshold of 36°C for the second heat activation, and the PE-free partially open state with a matched middle threshold of 46°C and a matched Ω_10_ of 3.85 for the second heat activation (17, 33, 34).

Finally, the thermoring-based systematic thermal instability (T_i_) in different gating states was also evaluated so that reasonable heat-evoked gating pathways of rTRPV2 were proposed to account for the use-dependent heat sensitization of rTRPV2 regardless of the accompanied rundown (17, 38). Taken together, the heat-induced gating states and pathways of rTRPV2 could be similarly defined by using the detergent-induced gating states once all the thermodynamic parameters aligned well theoretically and experimentally.

## Results

### Dynamic PE lipid at the vanilloid site in the resting closed state

**Figure 1:**
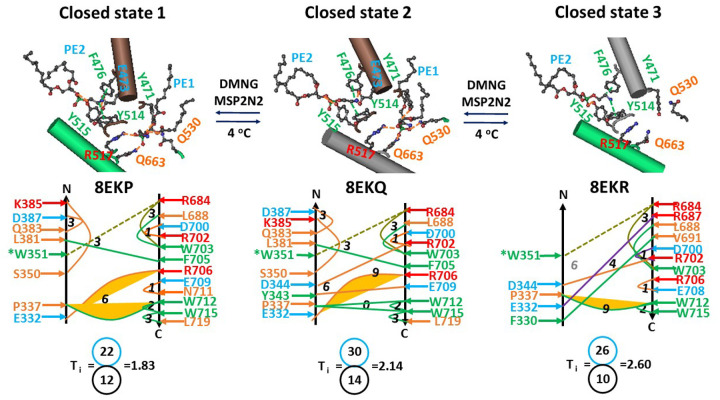
PE-dependent noncovalent interactions between C- and N-termini in closed rTRPV2. The homo-tetrameric cryo-EM structures of the rTRPV2 channels with or without PE bound at the vanilloid site and in VSLD in DMNG/MSP2N2 nanodiscs in three closed states at 4 °C (PDB ID, 8EKP, 8EKQ and 8EKR, respectively) were utilized for the model. Salt bridges, π interactions, and H-bonds between paired amino acid side chains are denoted in purple, green, and orange, respectively. The specific grid sizes necessary to regulate the least-stable noncovalent interactions in the grids are indicated with black numbers. The least-stable bridge in the biggest thermoring is highlighted in yellow. The total grid sizes and the total grid size-controlled noncovalent interactions between specific C- and N-termini are displayed in cyan and black circles, respectively, for the calculation of the local systematic thermal instability (T_i_).

The closed rTRPV2 channel exhibited three states when purified in the decyl maltose neopentyl glycol (DMNG) detergent and then reconsituted in membrane scaffold protein 2N2 (MSP2N2) nanodiscs at 4°C (35). A PE lipid in these three states was anchored by E473 and F476 on S3 and Y514 and Y515 on S4 in the VSLD via H-bonds and π interactions (Fig. 1). In addition, another PE lipid was present at the vanilloid site in states 1 and 2 but absent in state 3. It was stabilized by Y471 on the S2–S3 linker, R517 and Q630 on the S4–S5 linker, Q663 on the TRP domain through several H-bonds and the π interaction (Fig. 1). Therefore, the vanilloid site lipid is dynamic even in the resting closed state at low temperature. Given that the initial threshold (T_th_) for significant heat activation or differential scanning calorimetry (DSC) transition decreases from 48–52°C to 46°C for the rTRPV2/V1(357–434) chimeric channel (17, 36–38), it is necessary to examine if the noncovalent interaction between the C- and N- termini involves the initial T_th_ of 48–52°C.

### Identification of a pre-open closed state for initial heat activation

When the PE lipid was located at the vanilloid site in state 1 (PDB, 8EKP), there were 12 noncovalent interactions between the N-terminal segment F330-K385 and the C-terminal segment R684-L719. These noncovalent interactions formed a local grid-like mesh network. The biggest Grid_6_ was found to control the E332-R706 H-bond and the P337-W712 π interaction via a thermoring from E332 to P337, W712, N711, E709, R706 and back to E332. As the total number of grid sizes was 22, the local systematic thermal instability (T_i_) was calculated as 1.83 (Figure. 1).

In state 2 (PDB, 8EKQ), noncovalent interactions between the same N- and C-terminal segments increased to 14 and the biggest Grid_9_ was present to control the E332-R706 H-bond via a thermoring from E332, P337, W712, R706, and back to E332. With the total number of grid sizes increased to 30, the T_i_ also increased to 2.14 (Figure. 1).

However, when the PE lipid was randomly released from the active vanilloid site in state 3 (PDB, 8EKR), noncovalent interactions between the same N- and C-termini significantly decreased to 10 and the total number of grid sizes also lowered to 26 concurrently. Thus, the local Ti increased to 2.60. Meanwhile, another biggest Grid_9’_ appeared to govern the P337-W712 π interaction via a thermoring from P337 to E332, R687, L688, W703, R706, E708, W712 and back to P337 (Fig. 1). Because this P337-W712 interaction was energetically equivalent to 2 basic H-bonds (2 kcal/mol), the calculated melting temperature threshold (T_m_) was about 56°C, which did not match the inital T_th_ of 48–52°C (17, 36–38). Therefore, it is necessary to examine if the biggest thermoring with the matched T_m_ exists in other domains.

**Figure 2:**
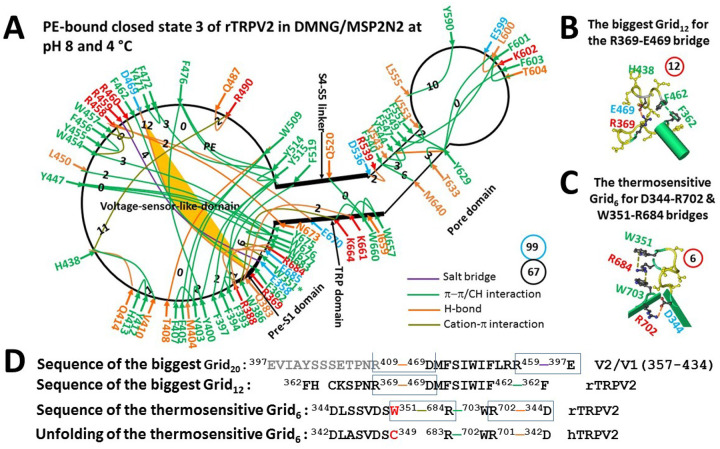
The grid-like noncovalently interacting mesh network along the PE-dependent gating pathway of the rTRPV2 channel in closed state 3 at pH8 and 4 °C. (A) The thermoring structure based on cryo-EM data of a single subunit of the closed state 3 of rTRPV2 in DMNG/MSP2N2 nanodisc at pH8 and 4 °C (PDB ID, 8EKR). The pore domain, the S4–S5 linker, the TRP domain, and the pre-S1 domain are indicated by black arrows, except for the VSLD. Salt bridges, π interactions, and H-bonds between paired amino acid side chains along the PE-dependent partial gating pathway from W351 to E685 are denoted in purple, green, and orange, respectively. The specific grid sizes necessary to regulate the least-stable noncovalent interactions in the grids are indicated with black numbers. The R369-E469 H-bond in the biggest Grid_12_ is emphasized in yellow. The total grid sizes and the total grid size-controlled noncovalent interactions along the whole PE-dependent gating pathway from F330 to W715 are displayed in cyan and black circles, respectively. (B) The structure of the biggest Grid_12_ with a 12-residue size to regulate the R369-E469 H-bond at the pre-S1/VSLD interface. (C) The structure of the putatively thermosensitive Grid_6_ with a 6-residue size to regulate the F344-R702 and W351-R684 bridges at the interface between C and N-termini. (D) The sequence of the biggest Grid_12_ to control the highlighted R369-E469 H-bond in the blue box. In contrast, the sequence of the putative biggest Grid_20_ in the rTRPV2/V1(357–434) chimeric channel to control the R409-D469 and R459-E397 bridges for a T_m_ of 34 °C. Additionaly, sequence alignment of the putatively thermosensitive Grid_6_ is shown between rTRPV2 and hTRPV2.

Since the release of PE from the active vanilloid site significantly increased the local systematic thermal instability (T_i_) from 1.83 or 2.14 to 2.60 (Fig. 1), a primary search began from the closed state 3. When the PE-dependent gating pathway extended to the peptide segment from W351 to E685, in addition to the W351-R684 cation-π interaction as shown in Fig. 1, fifty-seven additional noncovalent interactions formed a grid-like mesh network (Fig. 2A). Given that the total noncovalent interactions were 67 and the total grid sizes were 99 along the entire PE-dependent gating pathway from F330 to L719 (Figs. 1 & 2A; Table S1), the grid-based systematic thermal instability (T_i_) was about 1.48 (Table 1). This parameter was smaller than the T_i_ of 1.65 in PI-free rTRPV1 (28). Therefore, this closed state 3 was stable.

Notably, five π interactions linked Y447, L450 and W454 in the VSLD tightly with three aromatic residues Y676, W676 and W677 in the proximal C-terminal of the TRP domain. In addition, the S2–S3 linker (458–469) connected the TRP domain via the R458-E670/N673 H-bonds and the pre-S1 domain via the R459-E358 salt bridge, the F462-F362 π interaction and the D469-R369 H-bond. Finally, the H683-R388-E685 H-bonds, the W386-K664-W660-W657-K661 π interactions and the K661-Q383 H-bonds tightly connected the pre-S1 domain with the TRP domain and its proximal C-terminal region (Fig. 2A, Table S1). As a result, although the bigger Grid_11_ was observed to control the H438-R490 cation-π interaction in the VSLD along with the bigger Grid_10_ to govern the L555-Y590 CH-π interaction in the pore domain, the biggest Grid_12_ actually appeared at the pre-S1/VSLD interface. It had a 12-residue size to control the R369-D469 H-bond via a thermoring from F362 to R369, D469, F462, and back to F362 (Fig. 2B & 2D). Since the controled H-bond was energetically equivalent to 2 basic H-bond (2 kcal/mol), the calculated T_m_ was about 50°C, exactly matching the initial heat activation threshold of 48–52°C (17, 36–38) (Table 1). Therefore, the resting closed state 3 with PE still bound in the VSLD could serve as a pre-open closed for the first heat activation. In support of this proposal, the insertion of a serine residue at position 365 of rTRPV2 or the replacement of the segment 358–369 of rTRPV2 with the counterpart 397–409 of rTRPV1 in the rTRPV2/V1(357–434) chimeric channel decreases the threshold to 46°C for the dramatic heat activation or DSC transition possibly by changing the biggest Grid_12_ to Grid_13_ (Fig. 2A & 2D) (17, 31–33). Additionally, a small fraction of the F362-F462 π interaction may be broken in the rTRPV2/V1(357–434) chimeric channel. In this case, the biggest Grid_12_ would be subsituted by the biggest Grid_20_ (Fig. 2D). It had a 20-residue size to control the R409-D469 and E397-R459 salt bridges via a thermoring from E397 to R409, D469, R459, and back to E397. When two controlled bridges were energetically equivalent to 2 basic H-bonds (2 kcal/mol), the calculated melting temperature (T_m_) would be about 34°C, exactly matching the experimental threshold of 34.5°C for the small heat activation or DSC transition (36, 38).

### Identification of another pre-open closed state for the second heat activation

**Figure 3:**
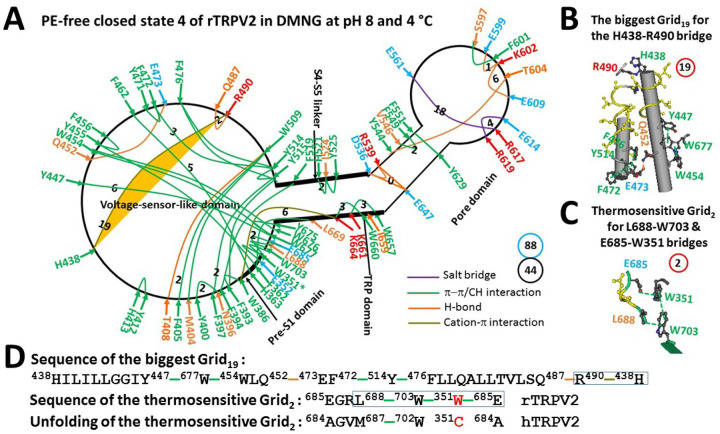
The grid-like noncovalently interacting mesh network along the PE-dependent minimal gating pathway of the PE-free closed state 4 of the rTRPV2 channel at pH8 and 4 °C. (A) The thermoring structure based on cryo-EM data of a single subunit of the PE-free closed rTRPV2 channel in DMNG at pH8 and 4 °C (PDB ID, 5HI9). The pore domain, the S4–S5 linker, the TRP domain, and the pre-S1 domain are indicated by black arrows, except for the VSLD. Salt bridges, π interactions, and H-bonds between paired amino acid side chains along the PE-dependent minimal gating pathway from W351 to W703 are denoted in purple, green, and orange, respectively. The specific grid sizes necessary to regulate the least-stable noncovalent interactions in the grids are indicated with black numbers. The H438-R490 cation-π interaction in the biggest Grid_19_ is emphasized in yellow. The total grid sizes and the total grid size-controlled noncovalent interactions along the PE-dependent minimal gating pathway are displayed in cyan and black circles, respectively. (B) The structure of the biggest Grid_19_ with a 19-residue size to regulate the H438-R490 cation-π interaction in the VSLD. (C) The structure of the putatively thermosensitive Grid_2_ with a 2-residue size to regulate the H438-R490 cation-π interaction in the VSLD. (D) The sequence of the biggest Grid_19_ to control the H438-R490 cation-π interaction highlighted in the blue box. Additionally, sequence alignment of the putatively thermosensitive Grid_2_ is shown between rTRPV2 and hTRPV2.

In addition to the PE-dependent closed state 3 in MSP2N2 nanodiscs, the fourth PE-independent closed state of rTRPV2 was also found in the presence of the DMNG detergent (33). The thermoring analyses showed that only 44 noncovalent interactions were identified along the PE-dependent gating pathway from W351 to W703 and none between the segments 330–350 and 704–719 (Fig. 3A; Table S2). When they formed a grid-like mesh network, the total number of grid sizes was 88. Therefore, the grid-based systematic thermal instability (T_i_) significantly increased from 1.48 to 2.00 in the absence of the MSP2N2 nanodiscs (Table 1).

Of special note, the least stable R369-D469 H-bond in the biggest Grid_12_ was disrupted by the DMNG detergent (Figs. 2A & 3A). Thus, the detergent may initially unfold the least-stable non-covalent interaction along the PE-dependent gating pathway from F330 to L719. As a result, a global conformational change occurred from the pre-S1/VSLD/TRP/pre-S1 interfaces to the S4–S5 linker/TRP interface and then to the pore domain. For example, at the pre-S1/VSLD/TRP/pre-S1 interfaces, the nearby E358-R459 salt bridge, the R458-E670/N673, K661-Q383, and H683-R388-E685 H-bonds, and the Y447-Y675/W676 and W676-L450 and F476-Y400 π interactions were disconnected. The nearby F362-F462 π interaction moved to the H363-F462 π interaction along with new noncovalent interactions such as the Y455-F362-L669 and W454-W677 π interactions and the Q452-E473 H-bond. Specifically, the disruption of the F476-Y400 π interaction dissociated the smallest Grid_0_ formed by the additional F476-Y515-Y400 π interactions. At the S4–S5 linker/TRP/VSLD/S4–S5 linker interfaces, the Q520-W660 π interaction was unfolded along with the related smallest Grid_0_ formed by the additional F519-F394 and F393-W386-K664-W660 π interactions. In the pore domain, the smallest Grid_0_ formed by the Y544-Y629 and E599-K602 H-bonds and the Y544-F601-T604 and L600-Y629-F603 π interactions was dissociated. Instead, another smallest Grid_0_ was created along with the formation of the D536-R539-E647-D536 H-bonds and the disruption of the nearby F540-M640 π interaction (Figs. 2A & 3A, Table S1 and S2).

Along with the rearrangement of the smallest Grid_0_, the biggest Grid_12_ in the initial pre-open state was replaced with the biggest Grid_19_ (Figs. 2A & 3A). It had a 19-residue size to control the least-stable H438-R490 cation-π interaction via a thermoring from H438 to Y447, W667, W454, Q452, E473, F472, Y514, F476, Q487, R490, and back to H438 (Fig. 3B & 3D). Since that controlled cation-π interaction was energetically equivalent to 2 basic H-bonds (2 kcal/mol), the calculated T_m_ was about 36°C, exactly matching the threshold of 35°C for the second heat activation (17). Therefore, the PE-free closed state 4 could act as another pre-open closed state for the second heat activation.

### Identification of the detergent-induced open state as the heat-evoked open state

**Figure 4:**
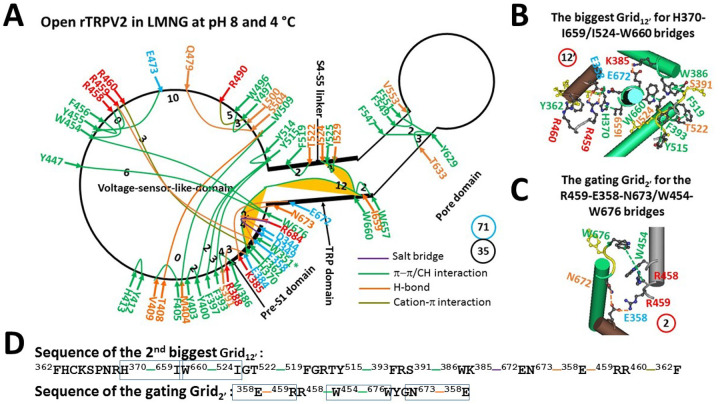
The grid-like noncovalently interacting mesh network along the PE-dependent minimal gating pathway of the PE-free rTRPV2 channel in an open state at pH8 and 4 °C. (A) The thermoring structure based on cryo-EM data of a single subunit of the open rTRPV2 channel in MNG at pH8 and 4 °C (PDB ID, 6BO4). The pore domain, the S4–S5 linker, the TRP domain, and the pre-S1 domain are indicated by black arrows except for the VSLD. Salt bridges, π interactions, and H-bonds between paired amino acid side chains along the PE-dependent minimal gating pathway from D344 to R684 are marked in purple, green, and orange, respectively. The specific grid sizes required to control the least-stable noncovalent interactions in the grids are labeled with black numbers. The H370-I659 and W660-I524 bridges in the biggest Grid_12’_are highlighted. The total grid sizes and the total grid size-controlled noncovalent interactions along the PE-dependent minimal gating pathway are shown in cyan and black circles, respectively. (B) The structure of the biggest Grid_12ʼ_ with a 12-residue size to control the H370-I659 and W660-I524 bridges at the pre-S1/TRP/S4–S5 linker interfaces. (C) The structure of the gating Grid_2’_ with a 2-residue size to control the R459-E358-N673 and W454-W676 bridges at the VSLD/pre-S1/C-terminus interfaces. (D) The sequence of the biggest Grid_12ʼ_ to control the H370-I659 and W660-I524 bridges highlighted in the blue boxes, and the sequence of the gating Grid_2ʼ_ to control the R459-E358-N673 and W454-W676 bridges highlighted in the blue boxes.

Like the only PE-free closed state in the DMNG detergent (33), the only PE-free open state was observed in the detergent lauryl maltose neopentyl glycol (LMNG) (34). When compared with two identified pre-open closed states, both the least-stable R369-D469 H-bond in the biggest Grid_12_ and the least-stable H438-R490 cation-π interaction in the biggest Grid_19_ were unfolded by the detergent in the same way as heat (Fig. 4A, Table S3). Instead, the new biggest Grid_12’_ was present at the pre-S1/TRP/S4–S5 linker/VSLD/pre-S1 interfaces to govern the least-stable H370-I659 and W660-I524 π interactions. It had a 12-residue size via a thermoring from F362 to H370, I659, W660, I524, T522, F519, Y515, F393, S391, W386, K385, E672, N673, E358, R459, R460, and back to F362 (Fig. 4B & 4D). Because two governed least-stable π interactions were energetically equivalent to 2.6 basic H-bonds (2.6 kcal/mol), the calculated T_m_ was about 56°C, matching the peak activity temperature of 56°C for the first and second heat activations (Table 1) (17, 36–38).

Along the PE-dependent gating pathway from F330 to L719, the total noncovalent interactions decreased to 35. When they formed a grid-like mesh network, the total grid sizes were 71, resulting in a systematic thermal instability (T_i_) of 2.03, similar to that in the second pre-open closed state (Fig. 4A, Table S3, Table 1). For the putative gating transition from the identified initial pre-open closed state to this open state, the calculated structural thermosensitivity (Ω_10_) was about 154.6, exactly matching the experimental functional thermosensitivity (Q_10_) of 154.7 (Table 1) (17). Accordingly, this PE-free open state could be used for the first heat activation. However, for the putative gating transition from the identified second pre-open closed state to this open state, the calculated Ω_10_ was about 14.8, which was inconsitent with the experimental Q_10_ of 3.88 for the second heat activation (17) (Table 1). Therefore, another intermediate was required.

### Identification of the detergent-induced partially-open state as the intermediate for the second heat activation

When the pore turret (564–589) was removed, a PE-free partially open state (PCD ID, 5HI9) was observed in the LMNG detergent (34). Since it had the similar disordered pore turret (558–594 vs 562–590) as the second pre-open closed state, it is interesting to investigate if it could be considered as the intermediate for the second heat activation.

**Figure 5:**
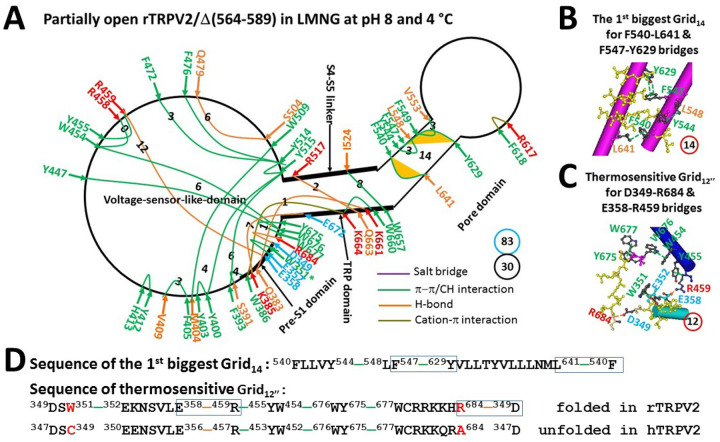
The grid-like noncovalently interacting mesh network along the PE-dependent minimal gating pathway of the PE-free rTRPV2 channel in a partially open state at pH8 and 4 °C. (A) The thermoring structure based on cryo-EM data of a single subunit of the partially open rTRPV2 channel in MNG at pH8 and 4 °C (PDB ID, 6BO5). The pore domain, the S4–S5 linker, the TRP domain, and the pre-S1 domain are indicated by black arrows except for the VSLD. Salt bridges, π interactions, and H-bonds between paired amino acid side chains along the PE-dependent minimal gating pathway from D349 to R684 are marked in purple, green, and orange, respectively. The specific grid sizes required to control the least-stable noncovalent interactions in the grids are labeled with black numbers. The F540-L641 and F547-Y629 bridges in the biggest Grid_14_ are highlighted. The total grid sizes and the total grid size-controlled noncovalent interactions along the PE-dependent minimal gating pathway are shown in cyan and black circles, respectively. (B) The structure of the biggest Grid_14_ with a 14-residue size to control the F540-L641 and F547-Y629 bridges at the S5/S6 interface. (C) The structure of the potentially thermosensitive Grid_12”_ with a 12-residue size to control the D349-R684 and E358-R459 bridges at the VSLD/pre-S1/C terminus interfaces. (D) The sequence of the biggest Grid_14_ to control the F540-L641 and F547-Y629 bridges highlighted in the blue boxes. Additionally, sequence alignment of the potentially thermosensitive Grid_12”_ is shown between rTRPV2 and hTRPV2.

The thermoring analyses indicated that the total noncovalent interactions decreased to 30 along the PE-dependent gating pathway from F330 to L719 (Fig. 5A, Table S4). When organized as a grid-like mesh network, the total number of grid sizes was 83, resulting in a grid-based systematic thermal instability of 2.77 (Fig. 5A, Table 1). For the putative gating transition from the second identified pre-open closed state to this partially open state, the calculated structural thermosensitivity (Ω_10_) was about 3.85, which closely matched to the expermental functional thermosensitivity (Q_10_) of 3.88 (Table 1). For the putative gating transition from this partially open state to the identified open state, the calculated (Ω_10_) was about 4.64, which also closely resembled the expermental Q_10_ of 3.88 (Table 1). Therefore, this partially open state could serve as the intermediate for the second heat activation.

Notably, as expected for the second heat activation, when the least stable H438-R490 cation-π interaction in the biggest Grid_19_ was disrupted, the biggest Grid_14_ was found in the pore domain (Figs. 3A & 5A). This Grid_14_ had a 14-residue size controlling the F540-L641 and F547-Y629 π interactions through a thermoring from F540 to Y544, F547, Y629, L641 and back to F540 (Fig. 5B & 5D). In addition to the traditional π–π interaction between the aromatic rings of F547 and Y629, the hydroxyl (-OH) group of Y629 also formed a random lone pair-π interaction with the aromatic ring of F547. This is reminiscent of the random N692-H410-N695 bridges for the irreversible inactivation of rTRPV1 (31).

Given that the second heat activation sometimes diverged between 46°C and 56°C (17), and the inactivated current around 55°C is comparable to the activated current around 46°C (38), whether the random Y629-F547 lone pair-π interaction is involved may define the partially open state as an inactivated state or an intermediate. When the controlled π interactions were energetically equivalent to 2.0 basic H-bonds (2 kcal/mol) in the absence of such a unique lone pair-π interaction, the calculated T_m_ was 46°C for the putative intermediate state. However, when the controlled π interactions were energetically equivalent to 3.0 basic H-bonds (3 kcal/mol) in the presence of such a unique lone pair-π interaction, the calculated T_m_ was 56°C for the putative inactivated state (Table 1). In any case, this partially-open state was unstable. When it converted to the final open state, the final maximal activity was close to the first heat activation (17).

### Role of intersubunit interactions in heat-evoked gating transitions

The pore turret (564–589) of rTRPV2 is disordered except in the open state. However, its removal locks the channel in the partially open state (34). On the other hand, the pore turret (604–626) of rTRPV1 is also unstructured (23). Deletion of its part (612–626) or full (604–626) fails to prevent heat activation (39–40). However, replacing the segment 613–626 with a poly glycine-based fragment of equal length inhibits the thermal response (41). Furthermore, rTRPV1 in MSP2N2 nanodiscs is heat-insensitive until capsaicin (Cap) outcompetes the PI lipid from the active vanilloid site (23). These observations are reminiscent of the regulatory role of the T1-T1 interface in the Kv4 gating (42–44). Given that no significant noncovalent interaction was observed between two turrets in the open and partially open states of rTRPV2 (Figs. 4A & 5A), it is necessary to investigate the intersubunit noncovalent interactions in these gating states.

**Figure 6:**
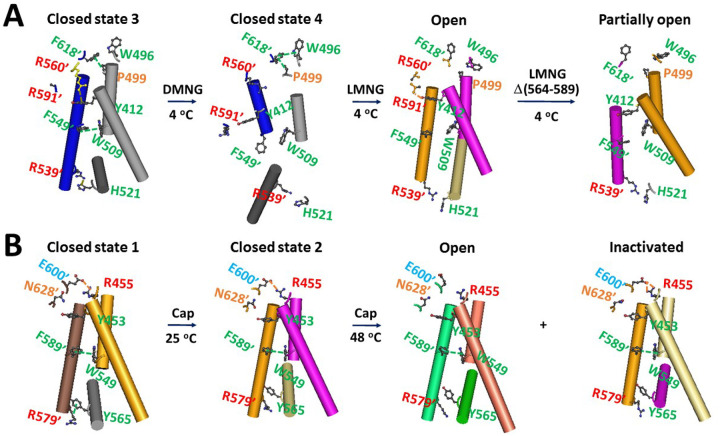
Comparison of lipid-dependent intersubunit interactions in different gating states of rTRPV2 (A) and rTRPV1 (B) at various temperatures. The detergent-induced cryo-EM structures of the PE-dependent rTRPV2 channel in the 3^rd^ and 4^th^ closed, open, and partially open states at 4 °C (PDB ID, 8EKR, 5HI9, 6BO4 and 6BO5, respectively) were used for model A. The capsaicin-induced cryo-EM structures of the PI-dependent rTRPV1 channel in the 1^st^ and 2^nd^ closed, open, and inactivated states from 4 °C to 48 °C (PDB ID, 8GF9, 7LPB, 7LPE and 7LPD/7LP9, respectively) were used for model B.

A recent study revealed that the intersubunit cation π interaction between H521 on the S4–S5 linker and R539’ on S5’ locks rTRPV2 in a closed state, and its disruption is required for channel activation (35, 45). Similarly, this swapping cation-π interaction was only found in the PE-bound closed state 3 of rTRPV2 in MSP2N2 nanodiscs, along with additional swapping W509-F549’ and Y412-R560’ and P499-W618’ π interactions between the pore domain of one subunit and the VSLD’ of the adjacent subunit (Fig. 6A). When the MSP2N2 nanodisc was removed, only P499-W618’ π interactions were observed in the DMNG detergent along with the W496-W618’ π interaction in the PE-free closed state 4 (Fig. 6A). Notably, Y412 on S1 from one subunit formed a swapping H-bond with the backbone NH at R591’ from the neighboring subunit in the open state rather than the partially open state in the LMNG detergent (Fig. 6A). Thus, such a tight swapping Y412-R591ʼ H-bond may allow H370 in the pre-S1 domain to be close enough to bridge I659 in the TRP domain for the stimulatory H370-I669 π interaction.

In contrast, Y565 on the S4–S5 linker of rTRPV1 also formed a similar swapping cation-π interaction with R579’ on S5’ in the PI-bound closed state at 4°C, along with the swapping W549-W589’ π interaction and R455-E600’ H-bond (Fig. 6B). This similar swapping cation/CH-π interaction also links the S4–S5 linker with S5’ in the closed state of TRPV3 or TRPV4 (24, 46). In the PI-free closed state at 25°C (PDB, 7LPB) and the PI-free open state at 48°C (PDB, 7LPE), the Y565-R579’ cation-π interaction was disrupted. However, in the PI-free inactivated state at 48°C (PDB, 7LPD), this swapping interaction was enhanced by bifurcated H-bonds (Fig. 6B). The similar case was also found in the PI-bound heat-insensitive inactivated state at 4°C (PDB, 7LP9). Since the swapping W549-F589’ π–π interaction was present in all these gating states, the tight swapping bifurcated R455-E600’ H-bonds may make the pore turret less exible and allow H410 or D411 in the pre-S1 domain to be close enough to bridge I696 and N495 in the TRP domain for the inhibtory H410-I696 and D411-N695 noncovalent interactions, preventing heat activation of rTRPV1 in MSP2N2 nanodiscs or in the G4PG4SG4S turret mutation (23, 28, 31, 41). This transmembrane gating regulation is also reminiscent of the external Zn^2+^-induced inhibition of the curcumin potentiation in the cysteine-engineered cystic fibrosis transmembrane conductance regulator (CFTR) mutants (47).

### W351-R684 cation-π interaction primes the heat activation of rTRPV2

The hTRPV2 channel is unresponsive to heat although oxidation induced small heat-evoked currents (2–3). A previous study suggested that both the N- and C-termini are essential for the heat sensation of rodent TRPV2 (2). Sequence alignment between rTRPV2 and hTRPV2 identified a critical W351-R684 cation-π interaction, which was present between the pre-S1 domain and the proximal TRP C-terminal domain in the identified initial pre-open closed state of rTRPV2 for the initial heat activation but disrupted by the corresponding residue C349 in hTRPV2 (W351 in rTRPV2) (Fig. 2C–D; Table S1). This strong interdomain bridge was controlled by the Grid_6_ via a thermoring from D344 to R702, W703, R684, W351 and back to D344 (Figs. 1 & 2C–D). Similarly, in the second identified pre-open closed state for the subsequent heat activation, E685-W351-W703 and L688-W703 π interactions formed a strong Grid_2_ via a thermoring from E685 to W351, W703, L688 and back to E685. However, this Grid_2_ would be disassociated in hTRPV2 (Fig. 3C–D). In the open state, the D344-R684 H-bond and the D349-R684 salt bridge were controlled by a smaller Grid_4_ via a thermoring from D344 to D349, T684 and back to D344. More importantly, the N673-E358-R459 H-bonds and the W454-W676 π–π interaction near W351 in rTRPV2 were governed by another smaller gating Grid_2ʼ_ via a thermoring from E358 to R459, R458, W454, W676, N673 and back to E358 (Fig. 4C–D; Table S3). Finally, in the partially open state, the second biggest Grid_12”_ was found to control the D349-R684 and E358-R459 H-bonds via a thermoring from D349, W351, E352, E358, R459, Y455, W454, E676, Y675, W677, R684 and back to D349 (Fig. 5C–D; Table S4). Given that the K425-E709 or K432-E704 salt bridge in rTRPV1 or mTRPV3 is equivalently missing in rTRPV2 when N673 replaces the equivalent K710 in rTRPV1 or K705 in mTRPV3 (28–29), all these W351-involved crtical interdomain interactions and small thermosensitive thermorings in four gating states may be required for heat sensing of rodent TRPV2 but unfolded by the cystein substitution in hTRPV2 (Figs. 1–5). Notably, when the membrane-proximal domain (MPD) (357–434) of rTRPV1 replaces the hTRPV2-MPD (317–390), although C390 in rTRPV1-MPD cannot form a π interaction with R683 in hTRPV2, two more residues ^361^EC^362^ in rTRPV1 may still relocate E391 in rTRPV1 in favor of a strong salt bridge with R683 in hTRPV2 for the high temperature sensitivity in the chimeric channel (36).

## Discussion

Not human but rodent TRPV2 has complex heat responses such as use-dependent sensitization accompanied by run-down. Although it has the highest initial temperature sensitivity (Q_10_ > 100), the initial high activation threshold (> 50°C) makes it challenging to capture the open state using the cryo-EM approach at or above 50°C. Therefore, traditional temperature titration is di cult to uncover the structural motifs for these complex heat responses (23–24). On the other hand, the cryo-EM structures of the mild detergent-induced closed and open states at 4°C have been available. Given that the thermoring basis for the heat sensing of TRPV1 and TRPV3 has been successfully confirmed by their cryo-EM structures at various temperatures (23–24, 28–29), thermoring analyses of these detergent-induced gating states were carried out in this study to define their roles in use-dependent heat sensitization of rTRPV2. Since the calculated melting temperature thresholds (T_m_) match the experimental threshold (T_th_) for each gating state with or without PE bound in the VSLD, and the calculated structural temperature sensitivity (Ω_10_) aligned well with the functional temperature sensitivity (Q_10_) between the specific closed and open states, these detergent-induced gating states could be used to reveal the thermoring basis for initial and subsequent heat responses of rTRPV2. Further thermostability analyses secured their distint gating pathways to be responsible for their complex heat responses.

### Thermoring basis for the initial thresholds for heat activations and DSC transitions

Recent thermoring studies have shown that the biggest Grid_13_ controls the least-stable Y401-R499 cation-π interaction at the pre-S1/VSLD interface. Its unfolding at the initial matched melting temperature threshold (T_m_) of 43°C is critical to release the PI lipid from the active vanilloid site for rTRPV1 activation by heat. On the other hand, the biggest Grid_14_ governs the least-stable E406-K504 salt bridge at the same pre-S1/VSLD interface with a matched T_m_ of 41°C for the initial heat activation of hTRPV1 with PI bound (28). When this initial T_m_ of 41–43°C aligns with the starting threshold of 42.7°C for the DSC transition of rTRPV1 (48), the heat-induced unfolding of the least-stable Y401-R499 or E406-K504 bridge is necessary for the heat activation of rTRPV1.

Once the PI lipid is released from the vanilloid site, the biggest Grid_21_ controls the least-stable Y401-R499 cation-π bridge, matching a T_m_ of 32°C to initiate the second heat activation of rTRPV1 (19, 28). Similarly, the biggest Grid_17_ dominates the R416-D519 salt bridge at the pre-S1/VSLD interface, matching a T_m_ of 40°C to activate oxidized mTRPV3 with the C612-C619 disulfide bond (29). However, in the reduced state, the biggest Grid_11_ controls the K614-N647 H-bond in the pore domain for the initial matched T_m_ of 52°C to activate mTRPV3 without the C612-C619 disulfide bond (29, 49). Since the heat unfolding of these least-stable noncovalent bridges in the various biggest thermorings has been confirmed by cryo-EM structures at or above the activation thresholds (T_th_) (23–24), the starting activation thresholds are determined by the least-stable noncovalent interactions in these biggest thermorings.

In this study, the biggest Grid_12_ was identified as controlling the least-stable R369-D469 H-bond at the pre-S/VSLD interface, resulting in an initial T_m_ of 50°C required to activate rTRPV2 with PE-bound in the VSLD (Fig. 2A, 2B & 2D, Table 1). Furthermore, the biggest Grid_19_ was found to govern the least-stable H438-R490 cation-π interaction, leading to a second T_m_ of 36°C necessary to activate PE-free rTRPV2 (Fig. 3A, 3B & 3D, Table 1). Since these calculated T_m_ values closely matched the experimental activation thresholds for the initial and second heat activations (17, 36–37), it is likely that the start activation thresholds are determined by the least-stable noncovalent interactions within these biggest thermorings. Supporting this idea, the matched T_m_ of 50°C for heat activation closely aligned with the start threshold of 47.7°C for the DSC transition of rTRPV2 (38). Therefore, the heat-induced unfolding of the least-stable R369-D469 and H438-R490 bridges was necessary for the first and second heat activations of rTRPV2, respectively.

### Thermoring basis for the end thresholds for heat activations and DSC transitions

Recent thermoring analyses also indicated that the biggest Grid_9_ in the heat-evoked open state of rTRPV1 controls the least-stable R557-E570 H-bond at the VSLD/S4–S5 linker interface for the maximum activity temperature of 56°C (28). This threshold of 56°C also corresponds to the end threshold of 56°C for the DSC transition of rTRPV1 (48), suggesting that the least-stable R557-E570 H-bond plays a key role in the heat activation of rTRPV1 (23, 28). Similarly, the biggest Grid_9ʼ_ in the heat-evoked open state of mTRPV3 regulates the least-stable D586-T680 and F590-L673 bridges for the maximum activity temperature of 61°C (29). Therefore, the least-stable noncovalent interactions in the biggest thermorings of the open channel are responsible for the maximum activity temperatures.

In this study, the two biggest thermorings were identified in the PE-free open or partially open state of rTRPV2. The biggest Grid_12’_ was used to control the least-stable H370-I659 and W660-I524 bridges at the pre-S1/TRP/S4–S5 linker interfaces for a maximum activity temperature of at least 56°C (Fig. 4A, 4B & 4D; Table 1). Alternatively, the biggest Grid_14_ was employed to govern the least-stable F540-L641 and F547-Y629 bridges in the pore domain for a maximal activity temperature of 46°C or 56°C in the intermediate or inactivated state (Fig. 5A, 5B & 5D; Table 1). Accordingly, these least-stable noncovalent interactions in these biggest thermorings may also be responsible for the maximal activity temperature of rTRPV2 (17, 36–38). Because the end threshold of 56°C for maximal heat activation also matched the end threshold of 56°C for the DSC transition of wild-type rTRPV2 (38), these least-stable H370-I659 and W660-I524 bridges were requred to secure the maximum activity temperature.

Notably, when the PI or PC lipid at the vanilloid site is released for heat activation of TRPV1 or TRPV3, the PI or PC lipid remains intact in the VSLD in the open state (23–24. 28–29). However, in the open or partially open state of rTRPV2, the PE lipid in the VSLD is also released (34). The same case was found in the activated rTRPV2 with 2-aminoethoxydiphenylborate (2-APB) and cannabidiol bound (45). In contrast, rTRPV2 remains closed at pH5 in the presence of a PE lipid in the VSLD even if the inhibitory swapping H521-R539ʼ cation bridge has been disrupted by the addition of the weak acid HOAc (35). Therefore, the release of both PE lipids at the vanilllid site and in the VSLD is required for rTRPV2 activation.

### Thermoring basis for the temperature sensitivity

Thermosensitive TRP channels are well-known for their high temperature sensitivity, known as Q_10_. When the structural thermosensitivity, referred to Ω_10_, is defined as a change in total grid sizes upon a change in total noncovalent interactions along the minimal lipid-dependent gating pathway, the calculated Ω_10_ values consistently match the experimental functional Q_10_. For example, Ω_10_ is 21.0 while Q_10_ is 21.9 for PI-bound rTRPV1. Once the PI is released from the active vanilloid site, Ω_10_ and Q_10_ are 15.9 and 16.4, respectively (23, 28).

The initial heat-evoked gating transition from the reduced closed state to the oxidized open state has a calculated Ω_10_ of 20.8, which closely matches the experimental Q_10_ of 20.6. In the second heat activation from the oxidized closed mTRPV3, the Ω_10_ and Q_10_ values are 2.69 and 2.32, respectively (24, 29). Hence, the temperature sensitivity is influenced not only by the heat-induced unfolding of noncovalent interactions but also by the simultaneous change in the total thermoring sizes along the minimal lipid-dependent gating pathway.

**Figure 7:**
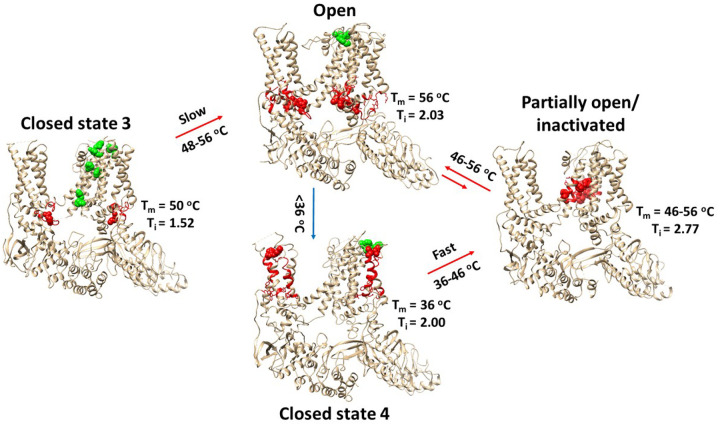
Temperature-dependent gating pathways of rTRPV2. The homo-tetrameric cryo-EM structures of rTRPV2 in the 3^rd^ and 4^th^ closed, open and partially open states in different detergents at 4 °C (PDB ID, 8EKR, 5HI9, 6BO4 and 6BO5, respectively) were used for the model. The partially open state also functions as a partially inactivated state. The corresponding biggest thermorings Grid_12_, Grid_19_, Grid_12’_ and Grid_14_ are colored red, respectively. All the least-stable noncovalent interactions controlled by those biggest thermorings are indicated by red space fill. The intersubunit interactions are indicated by green space fill.

In this study, the calculated Ω_10_ of 154.6 for the gating transition from the PE-bound closed state 3 to the PE-free open state was found be similar to the experimental Q_10_ of 154.7 for the initial heat activation (17) (Table 1). This suggests that this gating transition may be responsible for the very high thermosensitivity of the primary heat activation (Fig. 7). Similarly, the calculated Ω_10_ of 3.85–4.64 for the gating transitions from the PE-free closed state 4 to the PI-free partially open state, and then to the PE-free open state was similar to the experimental Q_10_ of 3.88 for the second heat activation (17) (Table 1). These two-step gating transitions may be responsible for the low thermosensitivity of the subsequent heat activation (Fig. 7).

Notably, the smallest Grid_0_ via the thermoring from Y400 to F476, Y514/Y515 and back to Y400, which is conserved in all gating states of rTRPV1 and mTRPV3 (28–29, 31), was disassociated in the PE-free closed state 4 and the open or partially open states (Figs. 2A, 3A, 4A & 5A). In this case, some smaller W351-involved thermorings at the pre S1/TRP/VSLD/pre-S1 interfaces in distinct gating states of rTRPV2 may serve as necessary anchors for heat activation of rodent TRPV2 (Figs. 1–5). However, the substitution of W351 by C349 in hTRPV2 may dampen these anchor thermorings and thus render hTRPV2 insensitive to heat (2–3).

### Thermoring basis for the complex gating pathways

Like TRPV3, rTRPV2 also exhibits use-dependent heat sensitization. In mTRPV3, the random formation of the disulfide bond between C612 and C619 in the pore domain upon the first heat activation decreased the threshold from 52°C to 30–40°C. In this case, repeated short heat stimuli allow more oxidized TRPV3 channels to increase the open probability at low temperatures, leading to use-dependent heat sensitization (26). Similarly, for rTRPV2, after the first heat activation at 53°C from the PE-bound closed state 3, the PE-free open state could not return to the PE-bound closed state 3 (Fig. 7). Instead, it may transition to the PE-free closed state 4 with a low T_m_ of 36°C. Thus, repeated short heat stimuli at 53°C would result in more PE-free closed state 4 to increase the open probability. Meanwhile, the open state may also transition to the partially inactivated state, which may be the reason why rTRPV2 exhibits concurrent rundown (33). However, the systematic thermal instability (T_i_) of the partially inactivated state was as high as 2.77. In contrast, the T_i_ values were 2.0 for the PE-free closed state 4 and the PE-free open state (Table 1). Therefore, despite the heat inactivation, use-dependent heat sensitization was still observed (Fig. 7) (17). In contrast, when the T_i_ of 1.04 in the inactivated state is lower than the T_i_ of 1.65 in the open state, rTRPV1 displays use-dependent desensitization (4; 19–20, 31).

### Intersubunit interaction rearrangements are coupled to heat activation

In addition to the rearrangement of the thermorings during heat activation, the intersubunit interactions were also rearranged for rTRPV2 and rTRPV1 similarly (Fig. 6). For example, the swapping of H521-R539’ or Y565-R579’ bridge at the interface between the S4–S5 linker and S5’ of rTRPV2 or rTRPV1 in the PE/PI-bound closed state was similarly disrupted in other PE/PI-free gating states. However, some differences in these swapping interactions were also observed between rTRPV2 and rTRPV1 (Fig. 6).

The first difference was that the W549-F589’ swapping interaction was present in all gating states of rTRPV1 but the equivalent W509-F549’ swapping bridge only appeared in the primary PE-bound closed state of rTRPV2. The second difference was that the P499-F618’ π interaction between S4 and the second pore turret’ was only present in the closed state of rTRPV2, while the similar R455-E600’ H-bond between S4 and the first pore turret’ not only appeared in the closed states but also enhanced in the inactivated state of rTRPV1. Most notably, the swapping Y412-R560’ π interaction in the closed state 3 and the Y412-R591’ H-bond in the open state of rTRPV2 were not observed similarly in rTRPV1 (Fig. 6). All these differences suggest that the pore turret may play distinct roles in heat-sensing of rTRPV1 and rTRPV2 (34, 39–40).

Notably, during the gating transition from the PE-bound closed state 3 to the PE-free open state, 32 noncovalent interactions were disrupted along the PE-dependent gating pathway from F330 to L719 (Table 1). Additionally, four intersubunit noncovalent interactions in the closed state 3 were disconnected in the PE-free open state, but the new swapping Y312-R591’ bridge was added (Fig. 6). Thus, the net decrease in total noncovalent bridges was 35 for each subunit of the homotetrameric rTRPV2 channel. Taking the average energy intensity of a noncovalent interaction as 1 kcal/mol, the total calculated activation enthalpy was 140 kcal/mol, approximately matching the experimental value of 145 ± 3.4 kcal/mol (38). In this regard, the cooperative intra-domain and inter-domain noncovalent rearrangements of the whole channel may be necessary for the heat activation of rTRPV2 once the least-stable noncovalent interaction along the lipid-dependent gating pathway from F330 to L719 is unfolded above the threshold of 50°C.

### Conclusions

Complex protein-lipid interactions in thermosensitive TRP channels present challenges in capturing the specific cryo-EM structures that encode their exact roles in sensing heat or cold. In this study, the highly sensitive thermoring model was utilized to redefine the available cryo-EM structures of rat TRPV2 in various detergent-induced gating states. Once the predicted thresholds, thermosensitivities and thermostabilities closely matched the experimental parameters, these structures could serve as a fundamental gating model for the intricate heat-sensing mechanism of rTRPV2.

## Methods

### Data mining resources

Three full-length cryo-EM 3D structures of the closed rTRPV2 channels with or without the PE lipid at the vanilloid site in DMNG/MSP2N2 nanodiscs at 4°C were examined as initial controls for heat activation (PDB ID, 8EKP for state 1, model resolution = 2.75 Å; 8EKQ for state 2, model resolution = 2.6 Å; 8EKR for state 3, model resolution = 3 Å, respectively) (35). In contrast, the full-length cryo-EM 3D structures of the open PE-free rTRPV2 channel and the partially open PE-free rTRPV2 without the pore turret (564–589) in the LMNG detergent at 4°C were studied to reveal the heat-evoked gating transitions (PDB ID, 6BO4, model resolution = 4 Å; 6BO5, model resolution = 3.6 Å, respectively) (34).

In addition, the full-length cryo-EM 3D structure of closed rTRPV2 with PE still bound in the VSLD in DMNG at 4°C was used as the fourth closed state for the second heat stimulation (PDB ID, 5HI9, model resolution = 4.4 Å) (33).

Finally, several cryo-EM 3D structures of temperature-gated rTRPV1 in MSP2N2 nanodiscs or hTRPV1 in cNW11 nanodiscs were also employed as controls to indicate the role of the pore turret in heat sensing of rTRPV2. These included closed PI-bound hTRPV1 at 4°C (PDB ID, 8GF9, model resolution = 2.58 Å) (50), PI-bound inactivated rTRPV1 at 4°C (PDB ID, 7LP9, model resolution = 2.63 Å), Cap-bound closed rTRPV1 at 25°C (PDB ID, 7LPB, model resolution = 3.54 Å), Cap-bound open rTRPV1 at 48°C (PDB ID, 7LPE, model resolution = 3.72 Å), and Cap-bound inactivated rTRPV1 at 48°C (PDB ID, 7LPD, model resolution = 3.55 Å) (23).

### Filtering noncovalent interactions

The stereo-selective and regio-selective inter-domain diagonal and intra-domain lateral noncovalent interactions along the PE-dependent gating pathway of rTRPV2 from F330 to L719 were analyzed using UCSF Chimera. The interactions were filtered by the same strict and consistent standard as previously used and confirmed (25–31). The examined noncovalent interactions included salt bridges, lone pair/CH/cation-π interactions and H-bonds between paired amino acid side chains. Specific cutoff distances and interaction angles for the different noncovalent interactions can be found in the online Supporting Information (Table S1, S2, S3 and S4).

### Mapping thermoring structures using graph theory

The study used the same protocol that had been previously described and validated to map the systematic fluidic grid-like noncovalent interaction mesh network as the thermoring structure (25–31). In this network, a topological grid was created with nodes representing amino acids and linked nodes representing noncovalent interactions along a single polypeptide chain. Graph theory and the Floyd–Warshall algorithm (51) were employed to determine the grid size as the shortest round path length to control the least-stable noncovalent interaction within the grid. The grid size also indicated the minimal number of side chains of free or silent amino acids that did not participate in any noncovalent interaction within the grid. Uncommon grid sizes were denoted in black numbers on the network map alongside the Grid_s_ with an s-residue size. The total numbers of noncovalent interactions (*N*) and total grid sizes (S) along the PE-dependent minimal gating pathway of rTRPV2 from F330 to L719 were calculated and displayed in black and cyan circles, respectively, next to the mesh network map for the calculation of the systematic thermal instability based on the equation as previously examined (25–31):

(1) $${\text{T}}_{\text{i}}=\text{S}/\text{N}$$

### Calculation of the melting temperature threshold for heat unfolding

The equation used to calculate the melting temperature threshold (T_m_) for the heat unfolding of the least-stable noncovalent interaction within a specific grid has been previously examined (25–31). It is as follows:

(2) $${\text{T}}_{\text{m}}\left({}^{\circ}\text{C}\right)=34+\left(\text{n}-2\right)\times 10+\left(20-\text{s}\right)\times 2$$

where, n represents the total number of basic H-bonds (~ 1 kcal/mol for each) that are energetically equivalent to the least-stable noncovalent interactions controlled by the given grid, and s is the size of the grid that controls the least-stable noncovalent interaction. Therefore, the heat capacity of the grid will increase as the grid size decreases or as the number of equivalent basic H-bonds increases.

### Evaluation of the systematic temperature sensitivity of rTRPV2

A gating transition of the thermosensitive TRPV1 or TRPV3 channel is always accompanied by a change in the energy density along the lipid-dependent minimal gating pathway (28–29). Accordingly, for enthalpy-driven activation of TRPV2 from a closed state within 10°C as a result of the broken biggest grid, if the chemical potential of a grid is theoretically defined as the maximal potential for equivalent residues in the grid to form the tightest β-hairpin with the smallest loop via noncovalent interactions (52), the grid-based structural thermo-sensitivity (Ω_10_) of a single ion channel for heat inactivation could be defined and calculated using the following equations as examined previously (28–29).

(3) $$\Omega_{10}={\left[\left({\text{S}}_{\text{c}}-{\text{S}}_{\text{o}}\right)\text{E}/2\right]}^{\left(\text{Hc}/\text{Ho}\right)}={\left[\left({\text{S}}_{\text{c}}-{\text{S}}_{\text{o}}\right)\text{E}/2\right]}^{\left[(\text{ENc}/\left(\text{ENo}\right)\right]}={\left[\left({\text{S}}_{\text{c}}-{\text{S}}_{\text{o}}\right)\text{E}/2\right]}^{\left(\text{Nc}/\text{No}\right)}$$

where, along the same defined PE-dependent minimal gating pathway of one subunit from F330 to L719, N_c_ and N_o_ represent the total noncovalent interactions, H_c_ and H_o_ denote the total enthalpy included in them, and S_c_ and S_o_ indicate the total grid sizes in the closed and open states, respectively. The energy intensity of a noncovalent interaction is denoted by E and is typically 1 kcal/mol. Thus, Ω_10_ factually reflects a thermo-evoked change in the total chemical potential of grids upon a thermo-evoked change in the total enthalpy included in the noncovalent interactions apparently from a closed state to an open or partially open state along the same defined PE-dependent minimal gating pathway of one subunit. For the gating transition from the partially open state to the final open state during the second heat activation, the partially open state replaces the closed state in the equations.

For a convenient comparison, the functional thermo-sensitivity (Q_10_) of a single ion channel for heat inactivation was calculated using the following equation:

(4) $$Q_{10}={\left({\text{X}}_{2}/{\text{X}}_{1}\right)}^{10/\left(\text{T}2-\text{T}1\right)}$$

where, X_1_ and X_2_ are the relative channel activity obtained at temperatures T1 and T2 (measured in Kelvin), respectively.

## Figures and Tables

**Table 1:** Comparison of cold-induced thermoring structural changes of rTRPV2 along the PE-dependent minimal gating pathway from F330 to L719. The comparative parameters are highlighted in bold.

PDB ID	6BO5	6BO4	5HI9	8EKR
**Construct**	Δ(564–589)	rTRPV2		
**Lipid in the VSLD**	free			bound
**Lipid at the active vanilloid site**	free			
**Lipid environment**	MNG		DMNG	DMNG/MSP2N2
**Sampling temperature, °C**	4			
**Gating state**	Partially open/inact	Open	Closed state 4	Closed state 3
**# of the biggest Grid**_**s**_	Grid_14_	Grid_12’_	Grid_19_	Grid_12_
**grid size (s)**	14	12	19	12
**The weakest noncovalent links in Grid**_**s**_	F540-L641 F547-Y629	H370-I659 I524-W660	H438-R490	R369-E469
**# of basic H-bonds (n) energetically equivalent to the least-stable noncovalent bridge**	2.0/3.0	2.6	2.0	2.0
**Total non-covalent interactions (N)**	30	35	44	67
**Total grid sizes (S), a.a**	83	71	88	99
**Systemic thermal instability (T**_**i**_ **)**	**2.77**	**2.03**	**2.00**	**1.48**
**Calculated T**_**m**_ **°C at E = 1 kcal/mol**	**46/56**	**56**	**36**	**50**
**Measured threshold T**_**th**_ **, °C**	**46/56**	**56**	**35**	**48–52**
**Calculated** Ω_**10**_**, mean at E = 1.0 kcal/mol**	**4.64**	**154.6**	**3.85**	**154.6**
**Measured Q**_**10**_	**3.88**	**154.7**	**3.88**	**154.7**
**Refs for T**_**th**_ **and Q**_**10**_	(17)	(17)	(17)	(17)

## Data Availability

All data generated or analysed during this study are included in this published article and Supporting Information.

## Abbreviations

ARD: Ankyrin repeat domain
Cap: capsaicin
cryo-EM: cryogenic electron microscopy
CFTR: cystic fibrosis transmembrane conductance regulator
DDM: n-dodecyl-b-D-maltoside
DMNG: decyl maltose neopentyl glycol
DSC: differential scanning calorimetry
LMNG: lauryl maltose neopentyl glycol
MDP: membrane-proximal domain
MSP2N2: membrane scaffold protein 2N2
PE: phosphatidylethanolamine
PC: phosphatidylcholine
PI: phosphatidylinositol
Ω_10_: structural thermosensitivity
Q_10_: functional thermosensitivity
T_i_: systematic thermal instability
T_m_: melting temperature threshold
T_th_: gating threshold
TRP: transient receptor potential
TRPV: TRP vanilloid
TRPVi: (i=1, 2, 3, 4)
hTRPV1: human TRPV1
mTRPV3: mouse TRPV3
2-APB: 2-aminoethoxydiphenylborate
rTRPV1: rat TRPV1
rTRPV2: rat TRPV2
VSLD: voltage-sensor-like domain
WT: wild-type
